# The Middlesex Hospital Laundry, Hendon

**Published:** 1912-11-16

**Authors:** 


### The Middlesex Hospital Laundry, Hendon.

BY THE SECRETARY-SUPERINTENDENT.

The following account deals exhaustively with the work and cost of a specially built and organised laundry where the whole of the hospital washing is done at an establishment entirely separate and distinct from the hospital proper:—

The laundry of the hospital was originally situated in the basement of the building, immediately under Bird Ward, an arrangement which, though by no means ideal, served its purpose for many years. With the growth of the hospital, however, the quantity of washing increased, and for the following reasons it became necessary in 1899 to make further provision for carrying out this work: (1) The washhouse was inconveniently situated, and the plant was too small; (2) the bleaching of the linen was poor; (3) the space taken up by the washhouse was urgently needed for other improvements to the hospital.

A freehold site, 350 feet by 125 feet, was acquired at Colindale Avenue, Hendon, for £700, and the present laundry buildings erected thereon at a cost of £9,992 13s. 6d. The machinery and boilers were installed by Messrs. Manlove, Alliott and Co., at a cost of £6,022 7s. 11d.; amounting to a total of £16,015 1s. 5d.

The buildings comprise: (1) Laundry; (2) engineer's cottage; (3) manageress' cottage; (4) motor shed; (5) drying ground.

The laundry contains: General workroom (drying room, ironing room, etc.); washhouse; receiving room; patients' distributing room; staff distributing room; boiler house; office; stores; workers' mess room and cloak room; women's lavatories.

The principal items of plant are: Two Cornish steam boilers; two duplex pumps for feeding boilers; one horizontal high-pressure non-condensing steam engine; two pairs of 59-inch rotary washing machines; four soap and soda dissolvers; two copper clothes boilers, each of 80 gallons capacity; two nests, of three each, ordinary glazed earthenware washing troughs; two 42-inch hydro extractors.

The hot-air apparatus in connection with the drying room consists of two heaters and two sets of india-rubber squeezing rollers; two blueing and clear rinsing machines; two ironing machines, "Great Western type," heated by steam entering at two places; two starching machines; two box mangles; two body linen ironers; one cuff and collar machine; sixteen gas-heated irons; one cold-water tank, capacity 1,500 gallons; one hot-water tank, capacity 1,000 gallons; four radiators for heating receiving room, packing room, and office; tables, racking, skirt boards, clothes boards, and clothes wagons.

When the laundry was opened a contractor was engaged to carry the linen backwards and forwards; but this arrangement proved too costly and was, moreover, inconvenient owing to the unpunctuality of the service.

A steam motor lorry was purchased from Messrs. Thornycroft in 1903 at a cost of £600, and remained in use until the commencement of the present year, when a Milnes-Daimler motor lorry to carry two tons was purchased at a cost of £710 14s., which sum, on the advice of the accountants, is to be spread over seven years.

The cost of running the steam motor, including driver's wages, was about £160. The cost of running the present motor cannot yet be given.

The motor makes two journeys a day (time about 50 minutes each way), except on Wednesdays, when it is cleaned and overhauled. On its first journey the motor brings clean linen to the hospital, arriving there at 9.30, and on the return journey it takes back soiled linen. A second delivery of clean linen is made at 4 P.M., and the motor then returns to the laundry with empty baskets.

The staff at the laundry consists of: One manageress, one engineer, one motor driver, two washhouse men, and from twenty to twenty-three female workers, who are, for the most part, engaged by the week, the number varying according to the amount of work to be done.

The average weekly amount paid in wages is £19 10s., exclusive of the salary of the manageress, who is a quarterly servant, and receives £86 13s. 6d. per annum, with house, fire, and light.

The workers commence at 7.45 A.M. daily, and finish at 7 P.M. On Saturdays the laundry closes at 1 o'clock. Time off: Lunch, 10 to 10.15; dinner, 1 to 2; tea, 4.30 to 5. Holidays: Men two weeks during the year, forewoman one week, all other women bank holidays.

The wages paid to the women are: Eighteen women and girls at wages ranging from 2s. 9d. to 5s. per day; three ironers on piecework, working three and a-half to four days a week, receive from 17s. to 20s. each.

The work is arranged as follows: Forewoman packs staff household linen; one packer and one sorter for nurses' and maids' linen; one sorter and one packer for wards; one girl for skirt machine; one girl for collar machine (fill in time on calender), one forewoman and four young girls on calender; four women for folding, mangling, and preparing; one woman (three days a week) for starching; one dryer, with occasional help from other hands, as all work that can possibly be dried on the drying ground is dried there, and this takes longer than it does to dry it in the drying closets.

Certain of the workers are transferred from department to department as the pressure of work demands.

The estimated number of pieces washed when the laundry was first built was 17,920 per week; the average number now is about 19,000. This includes washing from the Cancer and Barnato-Joel Charities, for which a separate financial account is kept. The sisters and nurses are allowed to send thirty articles weekly to the wash, and maids twenty. (These numbers do not include dressing gowns or jackets, blouses or frilled petticoats, which must be sent to a private laundry.)

Included in the number sent are several very large articles. Taking a typical week the number of large articles sent was as follows: 1,531 sheets, 2,383 draw sheets, 167 blankets, 234 quilts. The cost, including the amount written off for depreciation of plant and buildings, works out at considerably less than 1d. per piece.

It is a great advantage for a hospital, especially one in the heart of London, to have its laundry away from the general buildings. When the only space for this purpose at the disposal of the authorities is a portion of the basement immediately underneath the wards, as was the case at the Middlesex Hospital, the wisdom of such an arrangement is even more marked.

Experience has shown also that when the linen has to be despatched at stated times for the laundry greater promptness is shown in its collection from the various departments, whereas, on the other hand, when the laundry is on the premises there is tendency towards unpunctuality, both in collection and delivery, which involves much trouble to correct. Again, with the laundry away from the hospital a separate staff is necessary, and the duties of each member are carefully laid down, and there is no wastage of labour, as is frequently the case when the laundry hands, especially on the engineering side, are not definitely attached to that particular department.

*Middlesex Hospital, Washing Return,* 1910.

The following is the washing return for 1910. In 1911 the hospital was partially closed.

| | £ | s. | d. | £ | s. | d. | £ | s. | d. |
|---|---|---|---|---|---|---|---|---|---|
| Expenditure: | | | | | | | | | |
| Wages of laundry hands: | | | | | | | | | |
| Manageress ... ... ... ... ... | | | | 86 | 13 | 6 | | | |
| Mechanics ... ... ... ... ... | | | | 139 | 18 | 6 | | | |
| Menservants ... ... ... ... ... | | | | 98 | 18 | 0 | | | |
| Scrubbers ... ... ... ... ... | | | | 644 | 5 | 7 | | | |
| | | | | | | | 969 | 15 | 7 |
| Board: | | | | | | | | | |
| Materials and sundries (soap, soda, etc., brooms, brushes, utensils, overalls, clogs, baskets, etc.) ... ... ... | | | | | | | 203 | 8 | 8 |
| Renewal of furniture ... ... ... ... ... ... | | | | | | | 39 | 4 | 0 |
| Carriage ... ... ... ... ... ... ... ... | | | | | | | 88 | 12 | 4 |
| Fuel, power and light ... ... ... ... ... ... | | | | | | | 412 | 8 | 1 |
| Water ... ... ... ... ... ... ... ... | | | | | | | 114 | 15 | |
| Repairs to laundry building ... ... ... ... ... | | | | | | | 59 | 18 | 8 |
| Repairs to machinery and plant... ... ... ... ... | | | | | | | 28 | 11 | 6 |
| Insurance (fire and boiler) ... ... ... ... ... | | | | | | | 11 | 15 | 0 |
| Depreciation: | | | | | | | | | |
| Buildings: | £ | s. | d. | | | | | | |
| Original cost ... ... ... | 9,992 | 13 | 6 | | | | | | |
| Amount already written off ... | 449 | 13 | 3 | | | | | | |
| 1½ per cent. on ... ... ... | 9,543 | 0 | 3 | 143 | 2 | 11 | | | |
| Machinery and boilers: | | | | | | | | | |
| Original cost ... ... ... | 6,022 | 7 | 11 | | | | | | |
| Amount already written off ... | 1,355 | 0 | 9 | | | | | | |
| 7½ per cent. on ... ... ... | 4,667 | 7 | 2 | 350 | 1 | 0 | | | |
| Interest on capital, £16,015 1*s.* 5*d.*, at 3 per cent. | | | | 480 | 9 | 0 | | | |
| Rent, taxes, rates ... ... ... ... ... ... ... | | | | | | | 92 | 5 | 3 |
| Amount actually expended ... ... ... ... ... | | | | | | | 2,020 | 14 | 9 |
| Estimated for depreciation ... ... ... ... ... | | | | | | | 973 | 12 | 11 |

Total cost of washing done at Hospital Laundry at Hendon... £2,994 7 8
Average No. of pieces washed weekly, 16,436. Total pieces washed, 854,672.
Cost per 100 pieces, 7·076s.

